# Roles of osteoprotegerin in endocrine and metabolic disorders through receptor activator of nuclear factor kappa-B ligand/receptor activator of nuclear factor kappa-B signaling

**DOI:** 10.3389/fcell.2022.1005681

**Published:** 2022-11-03

**Authors:** Luodan Zhang, Fa Zeng, Minmin Jiang, Maozhen Han, Binbin Huang

**Affiliations:** ^1^ Department of Nephrology, Anhui Provincial Children’s Hospital, Children’s Hospital of Anhui Medical University, Hefei, Anhui, China; ^2^ Shenzhen Longhua Maternity and Child Healthcare Hospital, Shenzhen, Guangdong, China; ^3^ Department of Occupational Health and Environmental Health, School of Public Health, Anhui Medical University, Hefei, Anhui, China; ^4^ Department of Maternal, Child and Adolescent Health, School of Public Health, Anhui Medical University, MOE Key Laboratory of Population Health Across Life Cycle, NHC Key Laboratory of Study on Abnormal Gametes and Reproductive Tract, Anhui Provincial Key Laboratory of Population Health and Aristogenics, Hefei, Anhui, China; ^5^ College of Life Science, Anhui Medical University, Hefei, Anhui, China

**Keywords:** nuclear factor kappa-B ligand, receptor activator of NF-κB, osteoprotegerin, osteoporosis, cardiovascular disease, gestational diabetes mellitus

## Abstract

Endocrine and metabolic diseases show increasing incidence and high treatment costs worldwide. Due to the complexity of their etiology and mechanism, therapeutic strategies are still lacking. Osteoprotegerin (OPG), a member of the tumor necrosis factor receptor superfamily, appears to be a potential candidate for the treatment of these diseases. Studies based on clinical analysis and rodent animal models reveal the roles of OPG in various endocrine and metabolic processes or disorders, such as bone remodeling, vascular calcification, and β-cell proliferation, through the receptor activator of nuclear factor kappa-B ligand (RANKL) and the receptor activator of NF-κB (RANK). Thus, in this review, we mainly focus on relevant diseases, including osteoporosis, cardiovascular disease (CVD), diabetes, and gestational diabetes mellitus (GDM), to summarize the effects of the RANKL/RANK/OPG system in endocrine and metabolic tissues and diseases, thereby providing a comprehensive insight into OPG as a potential drug for endocrine and metabolic diseases.

## Introduction

Endocrine and metabolic disorders have become global health challenges due to their complications in physiological and pathological processes in multiple endocrine and metabolic tissues, which are risk factors for many diseases, such as osteoporosis, cardiovascular disease, diabetes mellitus, and pregnancy complications (Khosla and Hofbauer, 2017; Schafer et al., 2017; Helfer and Wu, 2018). To date, some key signaling systems responsible for the regulation of endocrine and metabolic processes have been revealed. Among them, RANKL/RANK signaling is a critical participant in endocrine metabolism (Kiechl et al., 2013; Kondegowda et al., 2015).

RANKL, a transmembrane ligand belonging to the TNF superfamily, is a crucial regulator of bone resorption and is a type II transmembrane glycoprotein composed of 317 amino acid residues (Anderson et al., 1997). RANKL can bind to RANK on neighboring cells and activate NF-κB signaling to regulate biological processes, such as the cell cycle, proliferation, differentiation, and apoptosis (Blomberg Jensen et al., 2021). RANKL is involved in regulating many processes, such as immune surveillance, bone resorption, monocyte chemotaxis, fibrosis, β-cell proliferation, and glucose homeostasis (Bernardi et al., 2016; Huang et al., 2020). RANKL can be found in membrane-bound and soluble forms, in which RANKL binds to two other RANKL molecules to form a trimer that binds to the receptor RANK (Rinotas et al., 2020). RANKL/RANK acts as a major regulator of BRCA1/BRCA2 mutations and is involved in controlling the development of breast cancer by inhibiting NF-κB activation and EGFR signaling (Galluzzi et al., 2016; Sirinian et al., 2018). RANKL mRNA in the bone marrow and lymphoid tissues with high expression, especially through the specific receptor RANK on osteoclast cells, promotes osteoclast maturation and inhibits osteoclast apoptosis. One study using a RANKL monoclonal antibody to block the specific binding of RANKL and RANK verified that the inhibition of osteoclasts could significantly increase the bone density of each place with a low bone mass after menopause and effectively reduce the conversion rate of bones (Kostenuik et al., 2009).

OPG acts as a soluble secreted glycoprotein first discovered by Simon et al. in the intestine of rats in 1997. OPG is a new member of the tumor necrosis factor receptor superfamily that can inhibit differentiation and maturation in osteoclasts and increase the bone density (Simonet et al., 1997). OPG is expressed in the healthy and pathological statuses of various tissues, including the bone, heart, blood vessels, kidney, liver, spleen, thymus, lymph nodes, placenta, adipose tissue, and pancreas (Shen et al., 2012; Bernardi et al., 2016). Indeed, OPG is a fusion receptor that selectively binds to RANKL and inhibits bone resorption, acting as a decoy receptor (Kwon et al., 1998; Akinci et al., 2011).

## Receptor activator of nuclear factor kappa-B ligand/receptor activator of nuclear factor kappa-B system regulates bone development

The bone is a special endocrine tissue that can protect the body organs and the hematopoietic bone marrow, providing structural support for muscles and maintaining the balance of ions and factors in vertebrates (Siddiqui and Partridge, 2016). The most striking function of the bone is paracrine signaling originating from bone cells. Current research has confirmed that the bone can be used as an endocrine organ (Zhou et al., 2021). The main components of a bone include osteoblasts, osteocytes, marrow adipocyte tissue, and mineralized fibrous tissue (Zhang et al., 2015; Lecka-Czernik et al., 2017). The RANKL/RANK/OPG system participates in pathological and physiological processes by secreting bone-derived hormones (Ferron et al., 2008; DiGirolamo et al., 2012; Wei et al., 2015; Zhang et al., 2015; Bernardi et al., 2016). RANKL can activate RANK in osteoclast precursor cells by residing in bone-forming cells and inducing osteoclastogenesis to promote bone resorption (Ikebuchi et al., 2018). Moreover, OPG acts as a natural inhibitor of RANKL and protects against bone loss in a mouse model (Simonet et al., 1997). OPG binds to RANKL and blocks the activation of RANK, which prevents bone resorption by differentiation and activation in osteoclasts (Ono et al., 2020). In clinics, there is a drug targeting RANKL, denosumab (Dmab), a monoclonal antibody that has been broadly used to treat osteoporosis and decrease the risk of fractures (McCloskey et al., 2012). Collectively, both osteogenesis and bone resorption are two key processes for bone remodeling, which is regulated by RANKL in osteocytes, suggesting that it is possible to use OPG as a natural biological inhibitor of RANKL for the treatment of bone-related diseases.

## Osteoprotegerin ameliorates cardiovascular disorders through the receptor activator of nuclear factor kappa-B ligand/receptor activator of nuclear factor kappa-B signaling

Cardiovascular diseases (CVDs) remain the leading cause of death and premature disability. In 2019, there were more than 523 million patients with CVD worldwide, of which approximately 18.6 million died from CVD (Roth et al., 2020). By 2020, cardiovascular diseases, such as atherosclerosis, had become the main cause of the total disease burden (Mozaffarian, 2016). These findings reinforce the need for diagnostic and prognostic tools that are helpful for CVD interventions. OPG levels were strongly correlated with arterial stiffness and the number of atherosclerotic sites in dysmetabolic patients (Monseu et al., 2016). Plasma OPG levels were increased in hypertensive patients compared to normal blood pressure volunteers, and serum OPG levels were significantly correlated with inflammation and hypertension, which may be used as a predictive indicator of hypertensive vascular endothelial dysfunction (Stepien et al., 2011; Arnold et al., 2019). Moreover, hypertensive patients with high plasma OPG levels are significantly more likely to have three or more target organ (cardiac, vascular, and renal) lesions at 10 years of cardiovascular risk for hypertensive retinopathy (Blazquez-Medela et al., 2012). These studies indicate that an increase in OPG levels might play a potential role in cardiovascular diseases. Recent research studies have indicated that vascular calcification is likely to be traditionally associated with the OPG/RANKL signaling system, which regulates bone remodeling. RANKL promoted vascular calcification, a disorder that causes blood vessel hardening and dysfunction (Lee et al., 2019), acting as a significant risk factor for type 2 diabetes mellitus, which is strongly associated with cardiovascular complications. Vascular calcification can be blocked by OPG acting as a RANKL decoy receptor (Ndip et al., 2011; Harper et al., 2016). An OPG-knockout mouse model showed aortic calcification, suggesting that OPG could protect large blood vessels from medial calcification (Callegari et al., 2013). These studies indicate that OPG could prevent vascular calcification.

In addition, experimental studies have shown that OPG can promote the adhesion of immune cells on endothelial cells, which is an important barrier to maintain the integrity of the inner surface of blood vessels, and its damage is the beginning of atherosclerosis, while the lack of OPG can cause endothelial cell damage (Rochette et al., 2018). With high OPG concentrations, endothelial cells stimulated by the vascular endothelial growth factor are more likely to survive. However, the mechanisms of OPG and RANKL remain to be determined and remain controversial in cardiovascular diseases. Hence, there might be an underlying mechanism involved in cardiovascular functions along with RANKL/RANK/OPG signaling.

## Osteoprotegerin affects glucose homeostasis

Diabetes results in insufficient absolute insulin secretion, resulting in higher blood glucose and causing metabolic disorders and even damage to multiple systems. Diabetes kills more than 3 million people worldwide each year, making it the third leading cause of death from cardiovascular diseases and tumors (Corona et al., 2011). Diabetes can be divided into type 1 and 2 diabetes, according to the etiology of diabetes. Moderate-type diabetes accounts for more than 90% of the total incidence. Insulin deficiency and insulin resistance are two indispensable causes of diabetes. In insulin-dependent diabetes mellitus, islet cells can secrete a certain amount of insulin, but the sensitivity of insulin is reduced, resulting in relative insulin deficiency in the body and causing metabolic disorders in the body and hyperglycemia. The main pathogenesis of diabetes is the inability of β-cells to secrete insulin required for metabolism, resulting in an imbalance in blood glucose homeostasis (Arnes and Sussel, 2015). Bone metabolism has received increasing attention regarding the regulation of blood glucose homeostasis. In a clinical study, an increase in serum OPG levels was reported in both patients with type 1 and type 2 diabetes mellitus compared to normal controls by different groups of investigators (Browner et al., 2001; Knudsen et al., 2003; Galluzzi et al., 2005; Giovannini et al., 2017). There is a strong potential association between OPG and diabetes. Rodent animal models reveal that OPG is a common gene upregulated in islets involved in β-cell replication from the models of β-cell expansion that include pregnancy, obesity, insulin resistance, and β-cell regeneration (Rieck et al., 2009). OPG regulated the proliferation of β-cells in young and old rodent models of diabetes mellitus through prolactin (Kondegowda et al., 2015). OPG-knockout mice and the inflammation induced by lipopolysaccharides (LPS) in a mouse model show an obvious decline in insulin secretion and impaired glucose tolerance. After the artificial addition of exogenous OPG, the symptoms are evidently improved, suggesting that OPG can increase the secretion of insulin, which regulates blood glucose homeostasis (Kuroda et al., 2016). In addition, in animal experiments, a high-fat diet can induce the upregulation of OPG levels in mice. In contrast, after exogenous injection of human OPG molecular protein, the blood glucose of mice is disturbed and their glucose tolerance is significantly impaired, resulting in impaired insulin secretion in mice (Toffoli et al., 2011; Bernardi et al., 2014). Human OPG induces inflammation in mice, leading to the death of cells, which has the opposite effect, with the consequence of disordered regulation of glucose metabolism (Kondegowda et al., 2015). Whether Dmab, as a targeted drug for osteoporosis, can control glucose remains to be deeply investigated in the future.

## Osteoprotegerin attenuated symptoms in gestational diabetes mellitus

Previously, different studies have elucidated the specific role of the RANKL/RANK/OPG system in mammary tissues, including gland physiology and hormone-driven epithelial proliferation during pregnancy (Infante et al., 2019). In addition, progesterone induces RANKL expression levels in both mice and humans (Liu et al., 2019), suggesting that RANKL might play roles in physiological processes during pregnancy. Regarding pregnancy complications, we mainly referred to hypertension and hyperglycemia during pregnancy. There was a significant increase in OPG during pregnancy, and the serum OPG rapidly decreased to the prepregnancy level after delivery, which is consistent with a previous experiment on mice (Yano et al., 2001; Naylor et al., 2003).

Pregnant women are first tested for impaired glucose tolerance and are diagnosed with GDM. Complications associated with gestational diabetes include overweight newborn infants or excessive babies, increased perinatal mortality, and risk of type 2 diabetes in offspring (Canouil et al., 2021). According to the International Diabetes Federation (IDF), the incidence of GDM has reached 16.8% in recent years. Retrospective clinical studies showed that the circulating levels of OPG in women with a history of GDM were significantly increased compared to normal women later in life, and they were positively correlated with various metabolic diseases with a high incidence later in life (Akinci et al., 2011; Telejko et al., 2015). We also have proven that placenta-derived OPG could regulate β-cell proliferation and glucose metabolism in placental glucose involved in glucose homeostasis in pregnant mice (Huang et al., 2020). OPG could promote glucose homeostasis during pregnancy, while there is no direct evidence to prove that OPG regulates hypertension during pregnancy. Taken together, these findings indicate that OPG seems to be a potential natural biology inhibitor for the treatment of pregnancy complications.

## Conclusion and prospects

**FIGURE 1:**
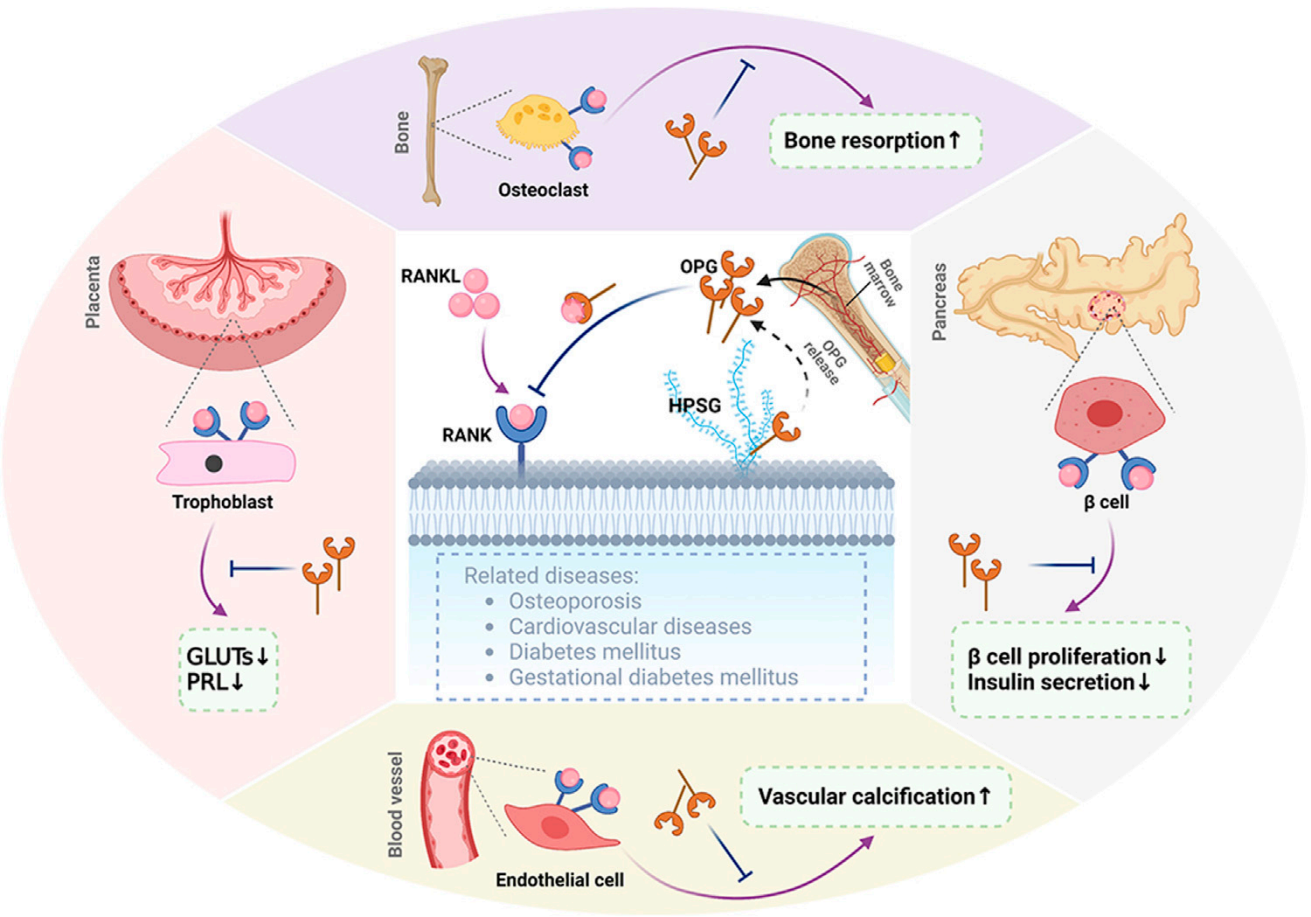
Representation of RANKL/RANK/OPG. RANKL binds to its receptor RANK, which can be found in membrane-bound molecules involved in bone resorption in osteoclasts, decreasing GLUT and PRL expression levels in the placenta, vascular calcification in blood vessels, and inhibiting β-cell proliferation and insulin secretion in islets. Osteoprotegerin (OPG), as a decoy receptor for RANKL, is secreted by the bone marrow and as a secreted glycoprotein. OPG competitively binds to RANKL to inhibit some functions of RANKL/RANK signaling in the bone, placenta, blood vessels, and pancreas. In addition, OPG binds to glycosaminoglycans such as heparin sulfate proteoglycans (HSPGs). OPG, osteoprotegerin; RANK, receptor activator of nuclear factor kappa-B; RANKL, receptor activator of nuclear factor kappa-B ligand; GLUTs, glucose transport proteins; PRL, prolactin. The figure was created with BioRender.com.

In summary, the RANKL/RANK/OPG system is important for homeostasis, CVD, bone diseases, and diabetes and has been one of the most important advances in physiology and biology in the past decade, as shown in Figure 1. The discovery of RANKL, RANK, and OPG has led to the development of specific inhibitors of RANKL, such as OPG, and a monoclonal antibody to RANKL has been tested in humans in clinical trials. Whether there are adverse effects on these tissues, especially on immune response, still needs to be clarified for the application of the RANKL/RANK/OPG system as a drug target.

## Data Availability

The original contributions presented in the study are included in the article/supplementary material. Further inquiries can be directed to the corresponding authors.
